# ADL recovery trajectory after discharge and its predictors among baseline-independent older inpatients

**DOI:** 10.1186/s12877-020-1481-8

**Published:** 2020-03-04

**Authors:** Xiuyue Li, Tingting Zheng, Yaqi Guan, Hui Li, Kexin Zhu, Lu Shen, Zhiqin Yin

**Affiliations:** Wenzhou Medical University, Facutly of Nursing, North near the intersection of Zhongxin North Road and Qiuzhen Road, Ouhai District, Wenzhou, 325000 Zhejiang province China

**Keywords:** ADL, Elderly hospitalised patients, ADL recovery, Predictive indicator

## Abstract

**Background:**

Among the previous studies about the ADL recovery and its predictors, the researches and resources used to study and protect the baseline-independent older patients from being permanently ADL-dependent was few. We aimed to describe the level of activities of daily living (ADL) at discharge and ADL change within 6 months after discharge in older patients who were ADL-independent before admission but became dependent because of acute illness, and to identify the predictors of early rehabilitation,so as to provide the basis to early intervention.

**Methods:**

Stratified cluster sampling was used to recruit 520 hospitalised older patients who were ADL-independent from departments of internal medicine at two tertiary hospitals from August 2017 to May 2018. Demographics, clinical data, and ADL status at 1, 3, and 6 months after discharge were collected. Data were analysed using descriptive statistics, Student’s t-test, Pearson’s chi-square test,Spearman’s correlation analysis, binary logistic regression analysis, and receiver operating characteristic (ROC) curve analysis.

**Results:**

There were 403 out of 520 patients completing the 6-month follow-up, and 229 (56.8%) regained independence at 6 months after discharge. There was an overall increasing trend in ADL with time. The recovery rate was the highest within the first month after discharge, gradually declined after 1 month, and changed less obviously from 3 to 6 months after discharge (*p* < 0.001). ADL score at discharge (OR = 1.034, *p* < 0.001), age (OR = 0.269, *p* = 0.001), post-discharge residence (OR = 0.390, *p* < 0.05), and cognition status at discharge (OR = 1.685, *p* < 0.05) were predictors of ADL recovery. The area under the curve of the four predictors combined was 0.763 (*p* < 0.001).

**Conclusion:**

Studying ADL recovery rate and its predicting indicators of the baseline independent inpatients at different time points provide a theoretical reference for the formulation of nursing plans and allocation of care resources.

## Introduction

Disability is a common health problem in the elderly that brings a heavy burden to individuals, families, and society. Acute admission and subsequent hospitalisation are important factors of disability among elderly persons. Many studies have shown that those patients with dependence in activities of daily living (ADL) persisting longer than 6 months rarely experience functional recovery [1–3]. Therefore, it is necessary to monitor the ADL changes of elderly patients after discharge. Brown et al. [4] found that about one-third of hospitalised patients over 70 years old had ADL dependence at discharge. Boyd et al. [5] followed up the recovery of ADL in older patients with acute illness for 1 year and found that those with new or additional ADL dependence at the time of discharge failed to regain independence, and the ability to recover in the months following discharge was related to their prognosis. Portegijs et al. [6] reported that a decline in function among older patients after 3 months of hospitalisation increased the risk of entering long-term care institutions within 1 year, regardless of whether their ADL level was damaged before admission. Huang et al. [7] assessed ADL changes in older inpatients from baseline to 3 months after discharge. Compared to the baseline level, the functional recovery of patients in the non-functional decline group showed a gradually recovered trend from admission to 3 months after discharge. However, the function of the older patients declined significantly after discharge in the functional decline group.

Although the above-mentioned studies examined the post-discharge function of older inpatients for a period of 1 month to more than 1 year, even 18 months, samples included older patients who were independent and dependent at the baseline. However, in order to prevent long-term dependence, it is more meaningful to examine ADL changes in older persons who are independent at baseline rather than including those already dependent. However, at present, few studies like this have been conducted. Hansen [8] and Lang et al. [9] investigated older patients who were ADL independent at baseline, but they followed them up for only 1 month. Bianca et al. [10] conducted a 1-year follow-up but only focused on the trajectory of functional changes and did not explore the influencing factors; in addition, the participants were seriously ill and dependent nursing home residents, and their ADL recovery was slow or even difficult. Therefore, their study has less effective reference value for older patients in general hospitals.

In consequence, we targeted older patients who were ADL-independent before admission and aimed to describe their ADL changes from admission to 6 months after discharge and identify the early stage of disease predictors of ADL recovery. This knowledge can provide a theoretical basis for the prevention of long-term dependence and the rational allocation of care resources in the future.

## Methods

### Setting and participants

**Fig. 1:**
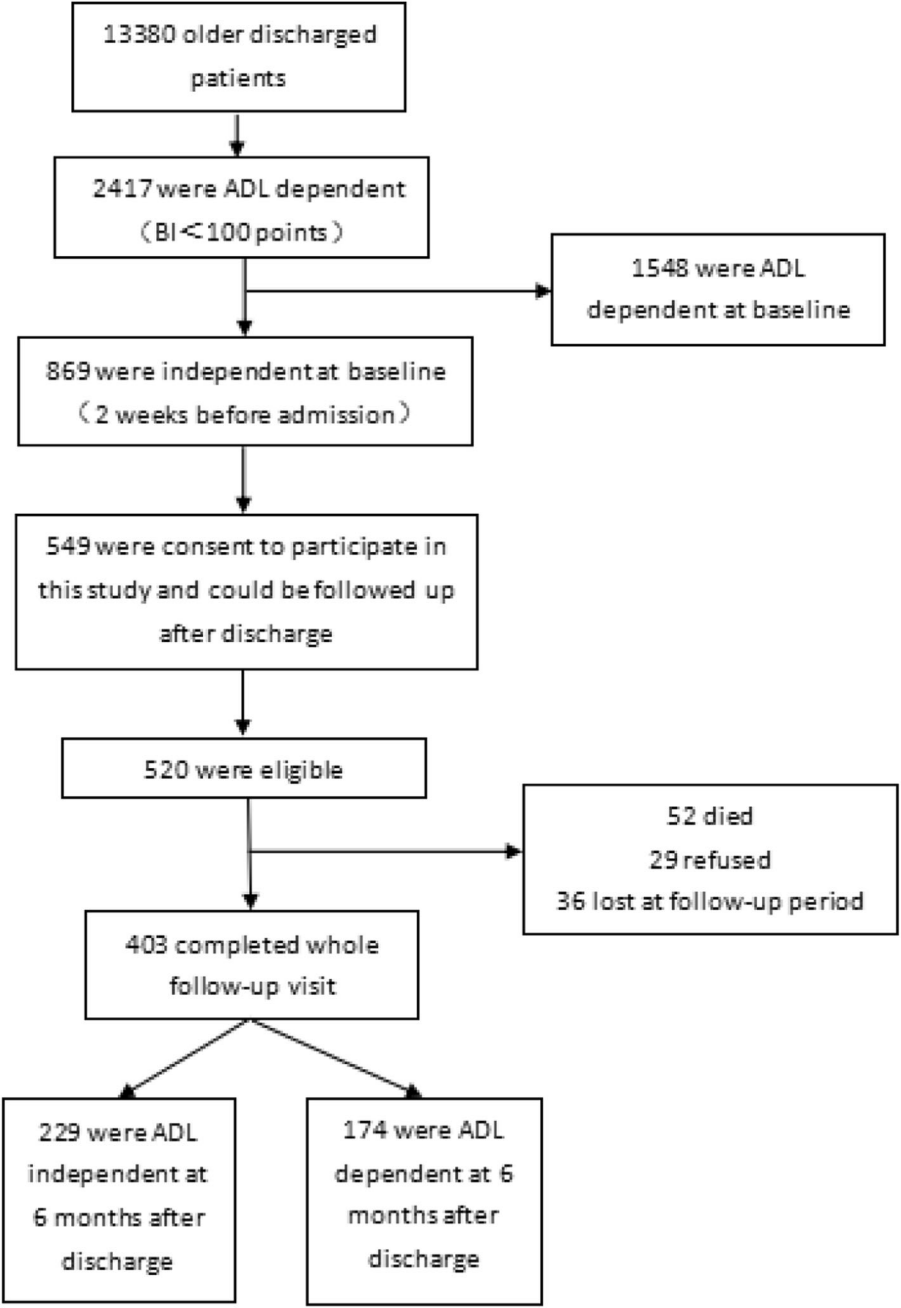
Sampling procedure

Stratified cluster sampling was used to recruit 520 hospitalised older patients from August 2017 to May 2018 from departments of neurology, cardiology, respiratory, geriatrics, and emergency at two Grade A tertiary hospitals in the central and southern regions of Zhejiang Province, China. Participants were patients aged 60 years or over who were independent at baseline (Barthel Index score = 100 points) and became dependent (i.e. Barthel Index score < 100 points) because of an acute illness or an acute attack of a chronic disease, and provided informed consent. Patients who had a length of stay under 48 h, were difficult to follow up, had an injury or surgical operation, or were critically ill were excluded. During the investigation period, a total of 13,380 older patients were discharged from the relevant departments of the two hospitals. There were 2417 (18.06%) ADL-dependent patients at the time of discharge, of which 1548 were dependent before admission, and 869 were independent before admission but dependent at the time of discharge (6.5% of the total cases and 7.9% of the baseline independent cases). Among the 869 cases, 549 questionnaires were sent out at the time of discharge, 520 were effectively responded, but only 403 cases completed the 6-month follow-up finally. Amongst them, 229 (56.8%) were independent in ADL after 6 months of discharge, and 174 (43.2%) were still dependent. The sampling procedure is detailed in Fig. 1.

### Research instruments

#### Patient information questionnaire

The Demographic Information Questionnaire was designed by the researchers and included sociodemographic data (e.g. age, gender, nationality, marital status, educational level, previous occupation, religious belief, type of health insurance, economic status, place of residence, pre-admission living style) and clinical information such as discharge diagnosis, therapeutic drugs during hospitalization, chronic diseases, laboratory indicators, and other disease data as well as body mass index (BMI) and other nutritional indicators.

#### Barthel index

The Barthel Index [11] includes 10 items (i.e. self-feeding, self-bathing, grooming, getting dressed, bowel control, bladder control, using the toilet, chair/bed transfer, mobility, and stairs climbing) and was used for ADL assessment in this study. According to the degree to which participants could complete the 10 activities, investigators gave 0, 5, and 10 points. Accordingly, the full score of 100 points implies ADL independence, and less than 100 points indicates ADL dependence. A score under 20 indicates extremely serious dependence, 25 to 45 severe dependence, 50 to 70 moderate dependence, and 75 to 95 mild dependence. The reliability of this test was 0.89, the interrater reliability was more than 0.95, and its validity was also good [12]. It can be used not only to evaluate the ADL status of patients before and after treatment, but also to predict therapeutic effect, hospitalisation time, and prognosis. It is the most widely used and studied ADL evaluation instrument.

#### Abbreviated mental test

The Abbreviated Mental Test includes 10 items; if answered correctly, each item is scored as 1 point; a total score of 8 to 10 indicates normal cognitive ability, less than 8 indicates cognitively abnormal [13].

#### Geriatric depression scale

The 5-Item Geriatric Depression Scale includes five yes/no questions, scored as 0 or 1 point; a score of 2 points or greater indicates a depression tendency; the sensitivity and specificity of the scale were 97 and 85%, respectively [14].

#### Instrumental activities of daily living

The Instrumental Activities of Daily Living (IADL) Scale is a self-reported instrument including eight items (i.e. meal preparing, cleanup, housekeeping, shopping, community mobility, telephone use, medication management, and money management).Level of IADL independence was measured by the number of IADLs requiring assistance.The full score is 8 (high function, independent), and the minimum 0 (low function, dependent) [15].

### Data collection

Investigators were uniformly trained research group members (two nursing postgraduate students from the central and southern regions of Zhejiang Province, China, who could speak local dialects). The investigators were led into the hospital by the head of each local hospital and entered the departments with the consent of the principal in charge. At the time of discharge or 1–2 days before discharge, after obtaining written informed consent from the patient or his/her family, data were collected.The baseline assessments (i.e. 2 weeks before admission) on ADL (Barthel Index) and IADL as well as the cognition status and depression at discharge were completed with the patients face to face.

The ADL assessment included patients’ ADL level 2 weeks before admission to determine whether they were ADL-independent at baseline (inclusion criteria), and the ADL level at discharge. Laboratory indexes and ADL scores at admission were obtained from the electronic medical records. Patients’ families or primary caregivers were interviewed when patients were unable or too ill to communicate (in this study, 6.2% of the respondents were surrogates).

ADL were assessed again by home visit or telephone follow-up at 1, 3, and 6 months after discharge. The duration of each assessment was 5–10 min. Since all patients were independent at baseline (i.e. 2 weeks before admission), we defined ADL recovery as a Barthel Index score of 100 points during follow-up. The study was approved by Ethics Committee of Wenzhou Medical University (Ethical certificate number: 2017–064).

### Data analysis

Continuous data were presented as mean ± SD if the variables had a normal distribution or similar, and Student’s t-test was used to compare the differences between groups. Otherwise, the median (1st quartile, 3rd quartile) was used. Categorical data were described as frequency (%), and Pearson’s chi-square test was applied to assess the differences between groups. Significant variables from the univariate analysis (*p* < 0.05) were then subjected to stepwise logistic regression analysis to evaluate the predictors of ADL independence. Spearman’s correlation analysis, binary logistic regression analysis, and ROC curve analysis were performed to identify the predictors and predictive value. For missing data, the mean substitution method was used for data interpolation(Additional file 1:Table S1). All analyses were carried out using Epidata 3.1 and SPSS 14.0.

## Results

### Sociodemographic characteristics

The age range for the 403 patients who completed the study was 60–92 years, and the mean age was (74.21 ± 7.69) years. The number of males (209) was slightly higher than that of females (194). Other sociodemographic information was detailed in Table 1.

**Table 1:** Socio-Demographic Information of Participants

Variable	*N* (%)
Age	
<65	52(12.9)
≥ 65	351(87.1)
Gender	
Male	209(51.9)
Female	194(48.1)
Residence	
Village	235(58.3)
Cities and towns	168(41.7)
Marital status	
Married	320(79.4)
Unmarried or widowed	83(20.6)
Education	
Illiteracy	195(48.4)
Primary school	126(31.3)
Junior middle school	56(13.9)
High school and above	26(6.4)
Previous occupation	
Physical labor	335(83.1)
Brain work	68(16.9)
Religion	
No	257(63.8)
Have	146(36.2)
Type of medical insurance	
Agricultural insurance	266(66.0)
Urban medical insurance	76(18.8)
Free medical insurance	41(10.2)
Self-financing medical insurance	20(5.0)
Economic status	
low	61(15.1)
medium	262(65.0)
high	80(19.9)
Resident manner	
Live with spouse only	241(59.8)
Live with spouses and children	56(13.9)
Live with children only	41(10.2)
Live alone	58(14.4)
Other	7(1.7)

### Clinical characteristics

There were 403 patients with neurological, respiratory, and cardiovascular conditions and patients with neurological diseases accounted for the majority of admissions. Other diseases were digestive and urinary (2 cases (0.5%), respectively). As for BMI (BMI = weight (kg)/height (m^2^)), the range was 13.33–32.87 kg/ m^2^, and the mean was (22.71 ± 3.64). According to Chinese evaluation criteria [16], 247 cases (61.3%) had normal BMI. Other health conditions were detailed in Table 2.

**Table 2:** Clinical Data of Participants

Variable	*N*(%)/ *x̅ ± s*	Variable	*x̅ ± s/M(P*_25_, *P*_75_)
Disease type		Number of medicine	12.26 ± 5.08
Nervous system	334(82.9)	Number of chronic diseases	3.96 ± 1.88
Respiratory system	27(6.7)	Number of discharge diagnosis	7.01 ± 2.52
Cardiovascular system	38(9.4)	Laboratory index	
Others	4(1.0)	Albumin(g/l)	37.78 ± 4.54
Post-discharge residence		Hemoglobin(g/l)	125.91 ± 17.64
Home	349(86.6)	Total protein(g/l)	64.87 ± 6.08
Institution	54(13.4)	Lymphocyte count(10^9^/l)	1.46 ± 0.67
BMI		Total Cholesterol (mmol/l)	4.31 ± 1.06
Low weight	42(10.4)	Glycerin trilaurate(mmol/l)	1.30(0.96, 1.69)
Normal	247(61.3)	Blood glucose(mmol/l)	5.58(4.93, 6.65)
Overweight	86(21.3)	Erythrocyte sedimentation rate(mm/h)	19(10, 27)
Obesity	28(7.0)	Length of stay in hospital(day)	11(9, 15)

### IADL at baseline, cognition, and depression at discharge

Among those 403 hospitalised older patients who were ADL dependent, IADL scores ranged from 0 to 8 points, and the median was 7 (5, 8). The cognitive scores ranged from 0 to 10 points, with a mean of (7.58 ± 2.35). There were 161(39.9%) patients with abnormal cognition, and 242 (60.1%) patients with normal cognition. The depression scores ranged from 0 to 5 points, with a median of 0 (0, 1). A total of 35 (8.7%) patients had a depressive tendency, and 368 (91.3%) did not.

### ADL change

**Fig. 2:**
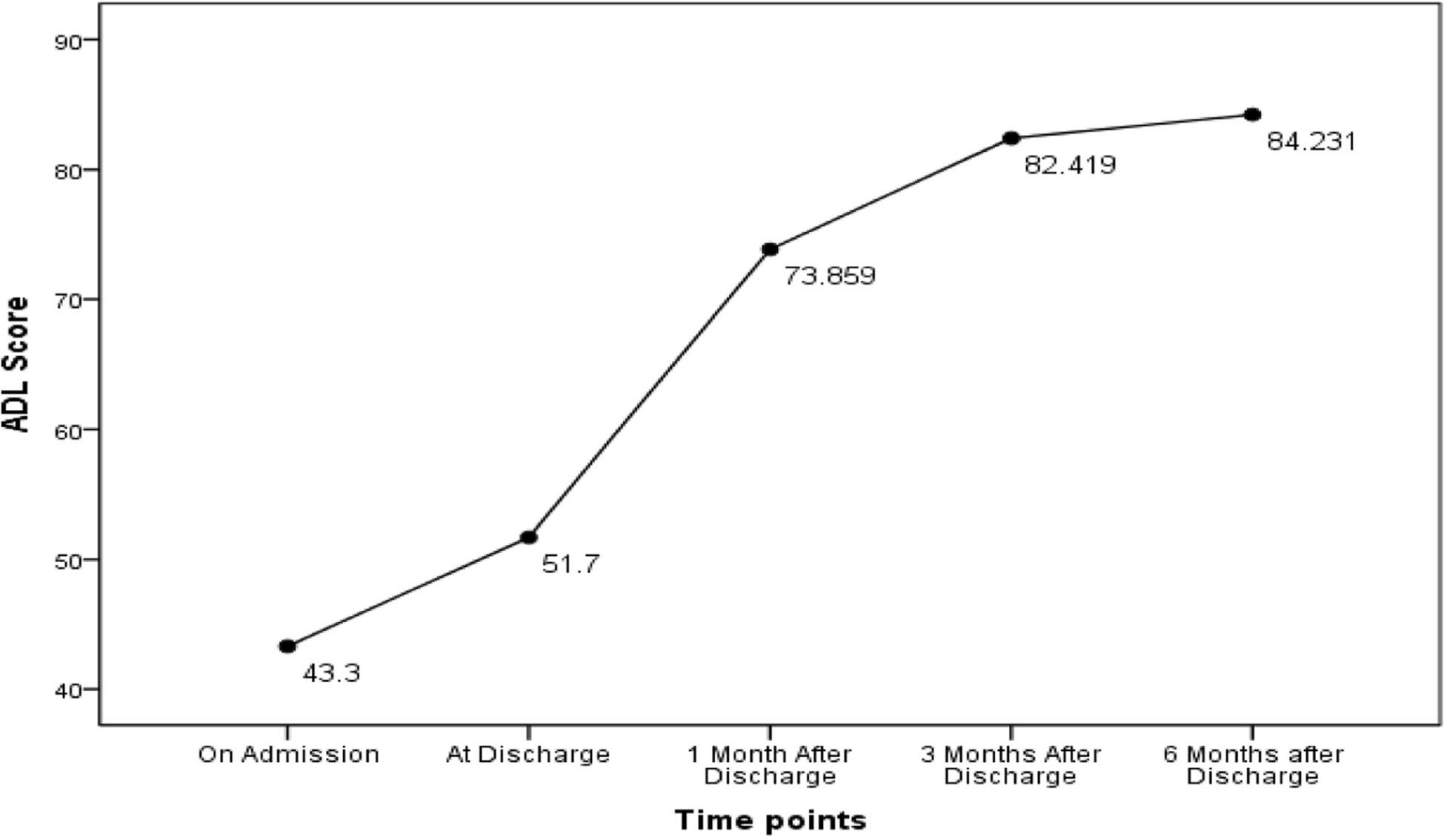
ADL Change Diagram of Participants

This study compared the ADL level at admission, discharge, and 1, 3, and 6 months after discharge. The ADL status of participants was presented in Table 3. Repeated measurement analysis showed that the difference in ADL scores at different time points from the time of admission to 6 months after discharge was statistically significant (*F* = 284.111, *p*<0.001). From the perspective of the change trend, the ADL of older patients showed an upward trend from admission to 6 months after discharge, with the recovery rate being highest within 1 month after discharge, and gradually slowing down after 1 month after discharge, and showing no obvious change from 3 to 6 months after discharge. The functional change diagram was shown in Fig. 2.

**Table 3:** ADL Status of Participants at each Time Point

Time point	Scoring range(point)	Median(point)	Dependent *N*(%)	Independent *N*(%)
ADL on admission	0~100	40(25, 60)	402(99.8)	1(0.2)
ADL at discharge	0~95	55(35, 70)	403(100.0)	0(0.0)
ADL at 1 month after discharge	0~100	80(60, 100)	285(70.7)	118(29.3)
ADL at 3 months after discharge	0~100	100(70, 100)	195(48.4)	208(51.6)
ADL at 6 months after discharge	0~100	100(100, 100)	174(43.2)	229(56.8)

### Univariate analyses of ADL recovery at 6 months after discharge

Taking ADL independence at 6 months after discharge (binary variable) as the dependent variable, univariate analyses were carried out with sociodemographic data, clinical data, pre-admission IADL score, ADL scores at admission, the depression score, cognition score and ADL scores at discharge as the independent variables. The results showed that age, post-discharge residence, number of medicine, glycerin trilaurate, blood glucose, length of stay, the cognition status, depression at discharge and ADL scores at admission and discharge had an significant effect on ADL independence at 6 months after discharge. The results are presented in Table 4.

**Table 4:** Univariate Analysis of ADL Recovery at 6 Months after Discharge

Variable	*N(%)/ x̅ ± S/M(P*_25_, *P*_75_)	At 6 months after discharge *N*(%)/ *x̅ ± S/M(P*_25_, *P*_75_)		*x*^2^*/t/Z*	*P value*
		Independent	Dependent		
Age					
<65	52(12.9)	39(75.0)	13(25.0)	8.040	0.005^**^
≥ 65	351(87.1)	190(54.1)	161(45.9)		
Post-discharge residence					
Home	349(86.6)	216(61.9)	133(38.1)	27.259	0.000^***^
Institution	54(13.4)	13(24.1)	41(75.9)		
Number of medicine	12.26 ± 5.08	11.74 ± 4.64	12.94 ± 5.55	−2.306	0.022^*^
Length of stay in hospital(day)	11(9, 15)	11(8, 15)	12(9, 16.25)	−2.278	0.023^*^
Glycerin trilaurate(mmol/l)	1.30(0.96, 1.69)	1.38(1.00, 1.825)	1.21(0.9075, 1.5625)	−2.265	0.024^*^
Blood glucose(mmol/l)	5.58(4.93, 6.65)	5.45(4.835, 6.485)	5.71(5.08, 6.713)	−2.253	0.024^*^
Cognitive status(point)					
<8	161(39.9)	70(43.5)	91(56.5)	19.463	0.000^***^
≥ 8	242(60.1)	159(65.7)	83(34.3)		
Depression	0(0, 1)	0(0, 1)	0.58(0, 1)	−2.510	0.012^*^
ADL scores at admission	40(25, 60)	45(30, 65)	35(20, 60)	−3.481	0.000^***^
ADL scores at discharge	55(35, 70)	65(50, 70)	45(25, 60)	−7.743	0.000^***^

^*^p<0.05; ^**^p<0.01; ^***^p<0.001; Factors that were not statistically significant were not listed

### Multivariate analysis of ADL recovery at 6 months after discharge

Taking ADL independence at 6 months after discharge as the dependent variable and the significant variables in univariate analyses as independent variables, binary logistic regression was carried out. The results showed that age < 65 years, returning home after discharge, the high cognitive scores and high ADL scores at discharge were significantly associated with ADL independence at 6 months after discharge. The results are presented in Table 5.

**Table 5:** Binary Logistic Regression Analysis of Independent predictors of Recovery at 6 Months after Discharge

	*B*	*S.E.*	*Wald*	*p value*	*OR*	95%*CI*	
						low	upper
ADL scores at discharge	0.034	0.006	28.475	0.000^***^	1.034	1.021	1.047
Age	−1.314	0.412	10.183	0.001^**^	0.269	0.120	0.602
Post-discharge residence	−0.943	0.413	5.210	0.022^*^	0.390	0.173	0.875
Cognition status	0.522	0.232	5.064	0.024^*^	1.685	1.070	2.654
Constant	1.269	1.140	1.238	0.266	3.557		

^*^p<0.05; ^**^p<0.01; ^***^p<0.001

### ROC curve for ADL Independence at 6 months after discharge

**Fig. 3:**
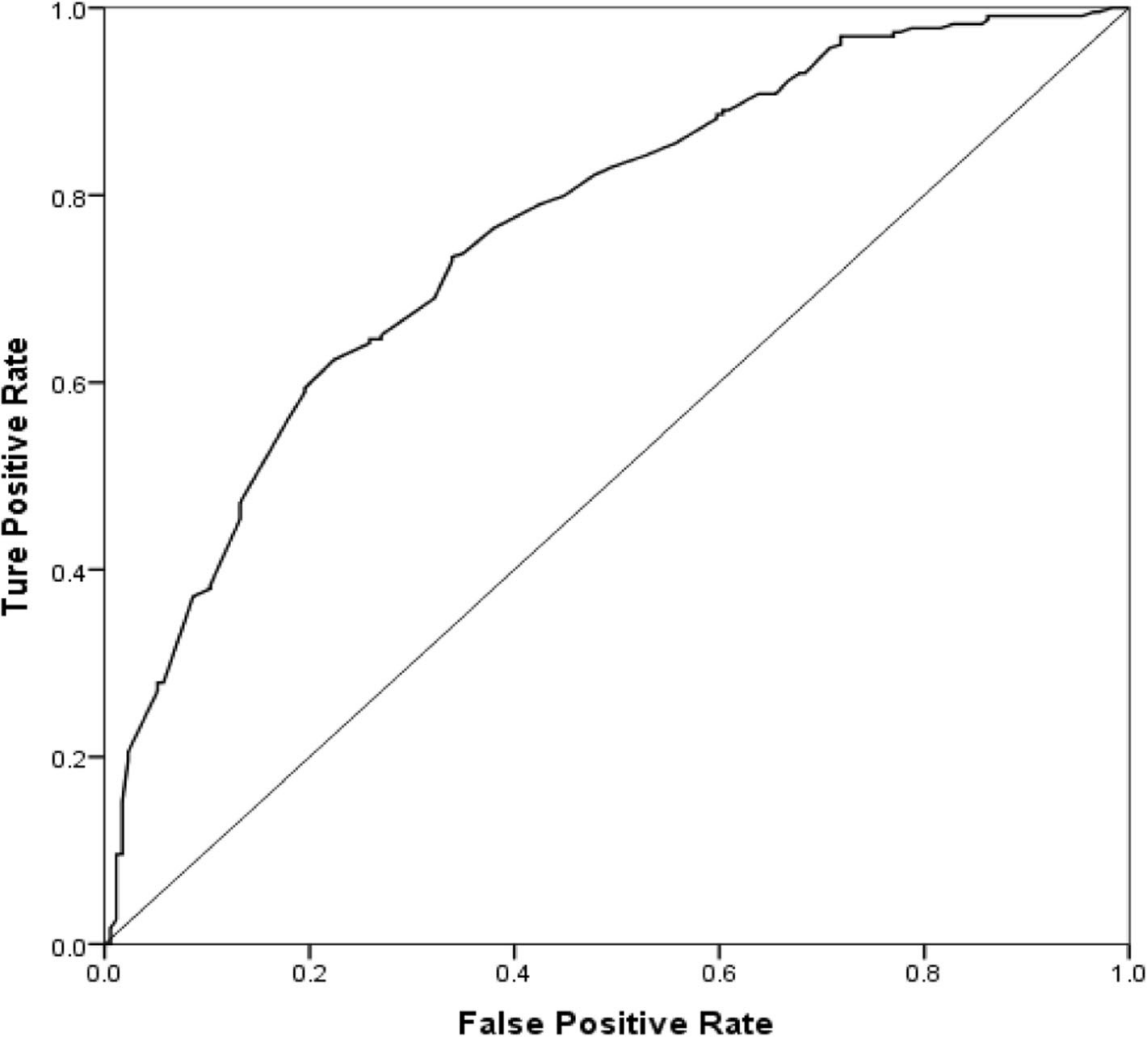
C-Index of ROC curves of ADL score at discharge. Footnote:Age, post-discharge residence and cognition predicted whether ADL recovered to independent in older patients at 6 months after discharge

In order to further examine the predictive value of the above factors for the recovery of ADL in older patients at 6 months after discharge, the prediction model was examined by ROC curve analysis. Taking ADL scores at discharge, age, post-discharge residence, and cognition scores at discharge as independent variables and ADL independence at 6 months after discharge as the dependent variable, the logistic regression analysis model was established, and the predictive efficiency of the ROC curve was fitted by the probability value in the model. The results showed that the area under the curve (AUC) for the four predictors combined was 0.763 (*p* < 0.001). These results are presented in Fig. 3.

## Discussion

Acute hospitalisation often causes a certain degree of damage to the abilities of daily living of elderly persons. The total number of admitted patients who were baseline-independent was 10,963 in the study period. Among them, 869 cases were ADL-dependent at discharge because of the acute illness, accounting for 7.9% of the total number of older patients discharged from hospital. This is similar to the results of the study conducted by Volpato et al. [17], indicating that acute hospitalisation can lead to ADL dependence in a considerably large number of independent elderly.

From the observation of ADL status, the proportion of older patients who were ADL dependent at 6 months after discharge was 43.3%, which was higher than that identified by Chen et al. [18]. Three-fifths of the patients included in Chen’s study were from surgical wards, while the patients in this study were all from internal medicine wards. Differences in diseases and treatments between internal and surgical departments may result in different recovery rates after discharge. In addition, Chen et al. excluded patients with cognitive impairment. But they were included in this study conversely. The adverse effects of cognitive impairment on the recovery of ADL after discharge have been reported in many studies [8, 19–21].

In this study, the rate of ADL-independent older patients at 6 months after discharge was 56.7% (i.e. proportion that returned to baseline), which was higher than the results of Boyd et al. [5]. The reason may be that Boyd et al. included older patients over 70 years old and patients were dependent at baseline, which is different from the present his study. Previous studies have shown that patients that are ADL-dependent at baseline may tend to be stable because of a long dependency time, which makes it less likely to restore ADL function [1, 3, 5, 18, 22, 23].

Similar to the results of previous studies [5, 7, 24], the ADL of hospitalised ADL-dependent older patients gradually recovered from admission to 6 months after discharge and the recovery rate was the highest at 1 month after discharge. From Fig. 2, the ADL level also increased to a certain extent from admission to discharge. This may be related to recovery from the acute illness, which is consistent with the results of Mudge et al. [25].

As for the factors related to functional recovery after discharge, previous studies had shown that age [7, 26, 27], cognition [8, 19–21], depression, albumin, and IADL at 2 weeks before admission affected ADL in older patients. Age and cognition were confirmed again in this study. However, some studies reported that age was not the most direct factor affecting ADL [28, 29], but these mainly involved short-term follow-ups. Hardy et al. [27] found that the effect of age on ADL was significant for a long time after becoming dependent, namely 6–12 months or longer after discharge. And older patients who had recovered independence at an early stage were prone to ADL dependence again over a long period of time. This is consistent with the results of the present study.

ADL score at discharge and post-discharge residence were predictors of ADL recovery in elderly patients at 6 months after discharge, which had not been reported in previous studies. The higher the ADL score of older patients at discharge, the greater the probability of ADL independence recovery at 6 months after discharge. Although previous studies had confirmed the correlation between physical function at discharge and prognosis [8, 30], ADL had not been used as an index to evaluate physical function, and Barthel Index had not been used to evaluate ADL. This result suggests that, first of all, clinicians should pay attention to the ADL recovery of older patients during hospitalisation. Despite the experience of functional loss, those whose function improved during hospitalisation were 2.3–2.9 times more likely to recover than those who continued to decline [31]. In addition, ADL evaluation should be considered as part of discharge assessment criteria. Furthermore, the post-discharge rehabilitation and nursing plans should be drawn up early, and medical and nursing resources should be reasonably allocated according to the possibility of ADL recovery.

This study also found that the ADL score at 6 months after discharge of older patients who returned home (87.64 ± 21.51) was significantly higher than that of those going to nursing homes or other care institutions (62.22 ± 31.31). It might be that patients going home could experience care, companionship, and support from their families, which might play a role in the recovery of their functions; or might be the ADL and diseases of the two above-mentioned groups were distinct at discharge. Patients who went to institutions after discharge had a score of (24.35 ± 19.35), which was significantly lower than that of those who went home after discharge (55.93 ± 19.44), which could be the reason why patients going home had a higher ADL score at 6 months after discharge.

Depression, albumin, and IADL 2 weeks before admission had no significant effect on the recovery of ADL at 6 months after discharge. In terms of depression, patients with a depressive tendency accounted for a small proportion of the sample (8.7%), which was lower than in previous surveys (17.3%) [32]. This may be related to the following reasons. Firstly, evaluation tools were different, and the 5-item Geriatric Depression Scale was used in this study, which is shorter than the tools in previous research. Secondly, evaluation time was different, as previous studies assessed depression during hospitalisation, while this study completed assessments 1–2 days before discharge or at discharge. Older patients who were about to be discharged might readjust their mental status, as the disease had improved and their environment would change soon. Thirdly, previous studies confirmed that cognitive impairment was associated with depression. However, in this study, patients with cognitive impairment accounted for a low proportion (6.2%). In terms of albumin, studies have shown that low albumin (< 35 g/l) was associated with ADL recovery. The proportion of patients with low albumin in this study was only 18.0%, which is much lower than in previous studies (76.7%) [5]. The reason may be that previous studies included populations that were dependent at baseline and whose basic nutritional status might be poor. However, the subjects in this study were independent at baseline, and most had good basic nutritional status and may not be prone to low albumin during hospitalisation. In addition, recent studies [21] pointed out that exploring the predictive effect of some biological parameters (e. g. albumin) on ADL in older patients were rare and some results of those studies were mutually contradictory [20, 33, 34]. Therefore, the relationship between albumin and ADL recovery after discharge in hospitalised ADL-dependent older patients needs to be further studied.

As for IADL 2 weeks before admission, some scholars held that patients who had much more IADL dependent were less likely to regain ADL independence. However, there was no association between the two in this study, which may need to be further examined in future research.

In this study, the ROC curve analysis of age, cognition, post-discharge residence, and ADL score at discharge showed that the AUC for the combined predictors was 0.763, which is higher than in previous studies (0.640–0.784) [35]. This indicates that the predictive value of the model is strong, which is helpful to predict the recovery of ADL of the elderly dependent population. Results of study indicate that the medical staffs should pay more attention to the assessments and individualized rehabilitation guidance among the patients who are greater than or equal to 65 years, with the AMT scores was below 8 and a lower score of ADL during the hospitalization. Additionally, enlarging the knowledge about rehabilitation and improving the skill to rehabilitate of the patients as well as their caregivers is essential to regain the ADL independence. Health care workers concentrate on the follow-up of patients and the rational care resources allocation after discharge, especially for the patients in the institution. Most importantly, continual assessments and rehabilitation guidance should be executed during the follow-up in this population.

### Study limitations

Due to practical limitations, this study could not examine other factors that may have an impact on ADL in early stages of disease, such as the comorbidity index. In addition, this study did not consider factors that may affect ADL after discharge in older patients, which need to be further studied in the future. It have been reported that patients with neurological diseases accounted for the majority of the hospitalized in this study, so the population of this study cannot represents all of acute hospitalized elderly patients.

## Conclusion

The ADL of hospitalised ADL-dependent older patients who were independent before admission showed a trend of gradual recovery within 6 months after discharge, but nearly half of the patients were still unable to regain ADL independence at 6 months after discharge. ADL score at discharge, age, post-discharge residence, and cognition were significantly associated with the recovery of ADL at 6 months after discharge in hospitalised ADL-dependent older patients, which could be used as a theoretical reference for the formulation of nursing plans and the allocation of care resources in the future.

## Supplementary information

**Additional file 1:.** Table S1 Description of Missing Data. For missing data, the mean substitution method was used for data interpolation. The missing data were detailed in Table S1.

## Data Availability

The datasets used and analysed during the current study are available from the corresponding author on reasonable request.

## Abbreviation

ADL: Activities of daily living
AUC: Area under the curve
BMI: Body mass index
IADL: The Instrumental Activities of Daily Living Scale
ROC: Receiver operating characteristic
