# Supplementary material for: ADL recovery trajectory after discharge and its predictors among baseline-independent older inpatients
Source: BMC Geriatr. 2020 Mar 4;20:86. doi: 10.1186/s12877-020-1481-8 (PMC7057590; doi:10.1186/s12877-020-1481-8)
Supplement: Supplementary file 1 — Additional file 1:. Table S1 Description of Missing Data. For missing data, the mean substitution method was used for data interpolation. The missing data were detailed in Table S1. [file 12877_2020_1481_MOESM1_ESM.doc]

**Table S1** Description of Missing Data

| Variable | Missing | Percent（%） |
| --- | --- | --- |
| Albumin | 4 | 1 |
| Hemoglobin | 1 | 0.2 |
| Total protein | 5 | 1.2 |
| Lymphocyte count | 1 | 0.2 |
| Cholesterol total | 10 | 2.5 |
| Glycerin trilaurate | 10 | 2.5 |
| Erythrocyte sedimentation rate | 46 | 11.4 |
| Blood sugar | 6 | 1.5 |
| Cognition | 11 | 2.7 |
| Depression | 35 | 8.7 |

Footnote: For missing data, the mean substitution method was used for data interpolation. The missing data were detailed in Table S1.
